# Psychotropic Taxonomies: Constructing a Therapeutic Framework for Psychiatry

**DOI:** 10.1016/j.biopsych.2024.12.004

**Published:** 2024-12-19

**Authors:** Robert A. McCutcheon, Philip Cowen, Matthew M. Nour, Toby Pillinger

**Affiliations:** 1 Department of Psychiatry, University of Oxford, Oxford, UK; 2 Oxford Health NHS Foundation trust, Oxford, UK; 3 Department of Psychosis Studies, Institute of Psychiatry, Psychology and Neuroscience, King’s College London, London, UK; 4 Max Planck UCL Centre for Computational Psychiatry and Ageing Research, University College London; 5 South London and Maudsley NHS Foundation Trust, London, UK

**Keywords:** Antipsychotic, Antidepressant, Precision medicine, Personalised medicine, Psychopharmacology, Drug, Treatment

## Abstract

Pharmacological interventions are a cornerstone of psychiatric practice. The taxonomies used to classify these interventions influence the treatment and interpretation of psychiatric symptoms. Disease-based classification systems (e.g., ‘antidepressant’ and ‘antipsychotic’) do not reflect the fact that psychotropic agents are used across diagnostic categories, nor account for the dimensional nature of both the psychopathology and biology of psychiatric illnesses.

In this review we discuss the history of psychotropic drug taxonomies and their influence on both clinical practice and drug development. We frame taxonomies as existing on a spectrum, with high-level disease-based approaches at one end and target-based molecular approaches at the other. Finally, we consider how data-driven methods might address the issue of classification at an intermediate level, based around transdiagnostic neurobiological and psychopathological markers.

## Introduction

Organizing an ever-expanding compendium of psychotropic substances has been recognized as a critical task, even before the dawn of modern psychopharmacology in the mid-twentieth century. How treatments are classified is closely linked to our understanding of the phenomenology and aetiology of patients’ complaints. Treatments may be developed to target a purported pathology, but a converse mapping may also develop, with mechanisms of efficacious treatments shaping models of disease pathophysiology. Indeed, psychiatric drug treatments have significantly influenced the evolution of biological approaches to understanding mental illness. The advent of modern psychopharmacology seven decades ago contributed to a neuroscientifically-informed reconceptualisation of mental illness that emphasised processes occurring at the level of neuromodulators (particularly monoamines).(1)

A classification scheme serves as a summary of the current state of scientific understanding, and plays a role in communicating this understanding and shaping of subsequent research. In the current paper we examine the evolution of psychotropic taxonomies and their influence on both research and clinical practice. We discuss the strengths and limitations of existing taxonomies, before considering what an improved taxonomy might look like.

### A History of Psychotropic Taxonomies

Prior to the modern era of psychopharmacology, attempts to formally categorise psychotropic compounds, such Louis Lewin’s 1931 volume ‘Phatastica’, approached classification from the perspective of subjective experiences and behavioural effects. Lewin proposed categories such as ‘euphoriants’, ‘hallucinating substances’, and ‘excitania’ i.e. stimulants.(2) The biomedical progress of the 20^th^ century was accompanied by a developing sense that physicians should not only match drug to disease, but also understand the pathophysiology of that disease and the mechanism by which the treatment acts.(3,4) This idea that physicians should be able to both explain and describe contributed to early debates as to whether a chemoreceptor-based or clinically-based approach to classifying psychotropic compounds was preferable.(4,5) Integrating these two approaches remains a challenge, in part owing to the multiple molecular effects of a single drug, and the complex relationship between receptor occupancy and downstream effects on cellular signalling, neural circuits, and behaviour.(6)

Professional bodies varied in their stance. In 1960, the American Medical Association recommended psychotropics be allocated to existing chemically defined categories based on pharmacology.(7) In contrast, a clinically-informed system might be expected to be more intuitive to practising physicians. However, even with a clinically-based approach, it was not clear how to precisely define the effects of the new compounds in patients. Initial terms to describe chlorpromazine and related compounds included ‘vegetative stabilisers’, ‘tranquillizers’, ‘ataractics’, ‘neuroleptics’, and ‘deturmoilizers’.(4) Jean Delay and Pierre Deniker, the psychiatrists who first characterised chlorpromazine’s clinical effects in psychosis, proposed a hierarchical symptom-based approach in which compounds were considered psycholeptics (reducers of emotional tension, including hypnotics, neuroleptics, and tranquilisers, expanding to include mood regulators in 1980), psychoanaleptics (elevators of mental tonus, including both stimulants such as amphetamines and mood stimulants such as monoamine oxidase inhibitors) and psychodysleptics (hallucinogens such as lysergic acid diethylamide (LSD)).(8,9) These taxonomies, while in some respects exemplifying a descriptive approach, still reflect underlying theoretical beliefs regarding mechanism, here tied to conceptions of psychic tension.

Delay and Deniker’s system formed the foundations of the World Health Organisation’s 1967 recommendations, which clearly linked compounds to disease states – themselves derived from consensus clinical taxonomies - with no regard to pharmacology: Neuroleptics, Anxiolytics, Antidepressants, Stimulants, and Hallucinogens.(10) Similarly, the 1968 British National Formulary (BNF) classified central nervous system drugs as analgesics, hypnotics, sedatives and tranquilisers, antidepressants, anticonvulsants, anti-Parkinsonian agents, cholinergic drugs, neuromuscular blocking drugs, and local anaesthetics. This scheme has changed little since, with the 2024 BNF listing hypnotics/anxiolytics, psychosis treatments, antidepressants, stimulants, obesity treatments, nausea treatments, analgesics, antiepileptics, parkinsonism, and substance dependence treatments.(11)

Refinements to this taxonomy that received meaningful adoption in clinical practice included subdivisions within the disorder-based categories. Following the approval of risperidone in the mid-1990s, a distinction between earlier antipsychotics (‘typical’ or ‘1^st^ generation’), and the more recently developed compounds (‘atypical’ or ‘2^nd^ generation’) emerged. This had substantial impact, entering clinical guidelines, and shaping practice to this day.(12) An assessment of the pharmacological and clinical evidence, however, does not support this form of dichotomisation as compounds in both groups overlap substantially in terms of both pharmacological and side-effect profile, with purported differences often exaggerated by higher doses employed in trials of older agents.(13) While the typical/atypical antipsychotic divide was primarily based on side-effect profile (14), with subsequent attempts to identify pharmacodynamic underpinnings (15), the dominant subdivisions within antidepressants reflect pharmacological mechanisms (e.g., selective serotonin reuptake inhibitors, monoamine oxidase inhibitors).

### Recent Approaches

The dominant, disease-based categorisation of psychotropics has remained relatively unchanged over the past 70 years. This reflects both its clinical utility, but also deficiencies in our understanding of both drug actions and disease pathophysiology. More recent approaches have attempted to move beyond this diagnosis-centred approach.

Schemes such as the Neuroscience Based Nomenclature (NbN) and others have attempted to incorporate pharmacodynamic properties into a classification scheme.(18,19) The NbN combines expert consensus with knowledge regarding the pharmacological properties of compounds, to provide both a neurochemically (e.g., dopamine, serotonin), and mode of action (e.g., antagonist) based categorisation. While more accurately reflecting compound pharmacology, there is still a necessary condensation of relevant pharmacodynamic data. Given the rich pharmacology of many psychotropics this has some undesirable consequences. For example, histaminergic affinities do not play a role in the classification of antipsychotics, despite the fact the histamine H1 receptor is central to both the side-effects and anxiolytic properties associated with these drugs.(20,21) Also the pharmacological properties relevant to psychotropic effects in humans may differ from that proposed on the basis of animal studies. For example, agomelatine is classified as a melatonin agonist and 5-HT2C receptor antagonist but whether agomelatine blocks 5-HT2C receptors at therapeutic doses in humans is doubtful.(22)

Another recent attempt at classification employed a more directly data-driven approach, and grouped antipsychotics based on binding affinities for 42 separate receptor types.(24) Antipsychotics were clustered according to the similarity of their receptor profiles. This identified four groups (Figure 2) which showed distinct clinical and pharmacodynamic profiles and did not map to the typical/atypical dichotomy. Limitations of this method include the fact that the unbiased approach means equal weighting is given to all aspects of the receptor profile. As a result, in cases where an individual receptor has a disproportionate impact on a compound’s clinical effect, the importance of that receptor may be lost in the analysis. Furthermore, the fact that some compounds are effective in several distinct clinical conditions, such as doxepin in the treatment of both urticaria and depression, underscores that it is the neurobehavioural signature of a compound in addition to its pharmacodynamic profile that is crucial to understanding its place in psychiatric treatment.

### Dimensions and Data

The drawbacks of a disorder-based classification partially reflect the limitations inherent to psychiatric diagnosis. Psychiatric diagnoses do not reflect discrete patterns of symptomatology. The same person can meet criteria for multiple diagnoses simultaneously, while two people with the same diagnosis can share no symptoms, phenomena that suggest an imperfect mapping between diagnostic entities and psychopathological processes.(25,26) Psychiatric symptoms such as anxiety and dysphoria are dimensional, varying along a severity spectrum from normal variation inherent to the human condition to profound functional impairment.(27) Unsurprisingly, dimensional models capture this more accurately than the expert-defined categorical schemes used in clinical practice.(28) Furthermore, psychiatric diagnoses do not reflect discrete patterns of neurobiology; while in some cases there is a clearly defined aetiology, in most psychiatric disorders the biological underpinnings also appear dimensional.(29,30) Genome wide association studies have illustrated that the genetic architecture of these disorders is best conceptualised as a spectrum of risk.(31–34) Similarly, neuroimaging studies (structural, molecular, and functional) show a lack of categorical boundaries both between diagnostic groups, and between patients and controls.(35–41)

Due to the symptomatic and neurobiological heterogeneity within disease categories, any classification scheme predicated on mapping between diagnosis and therapeutic effects of a compound will have inadequacies.(25) This was immediately recognised with the discovery of the first modern psychopharmacological treatments, with a view that a link to symptomatic effects, such as ‘mood lifting’, ‘inhibition reduction’, and ‘sedation’, might capture the drug effects more accurately than a diagnosis-based approach.(4) Recent analyses are consistent with this view, where considering compounds in light of their impact on symptom groups may characterise their effects more accurately than a diagnosis-based interpretation.(42) Inherent here, is a subtle tension between the ambitions of psychopharmacology that is more explicit in other areas of medicine: to alleviate symptoms, or to address causal mechanisms and modify disease. A middle-ground is also apparent given that in some models of mental disorder, symptoms and disease are not distinct entities.(43)

**Figure 1:**
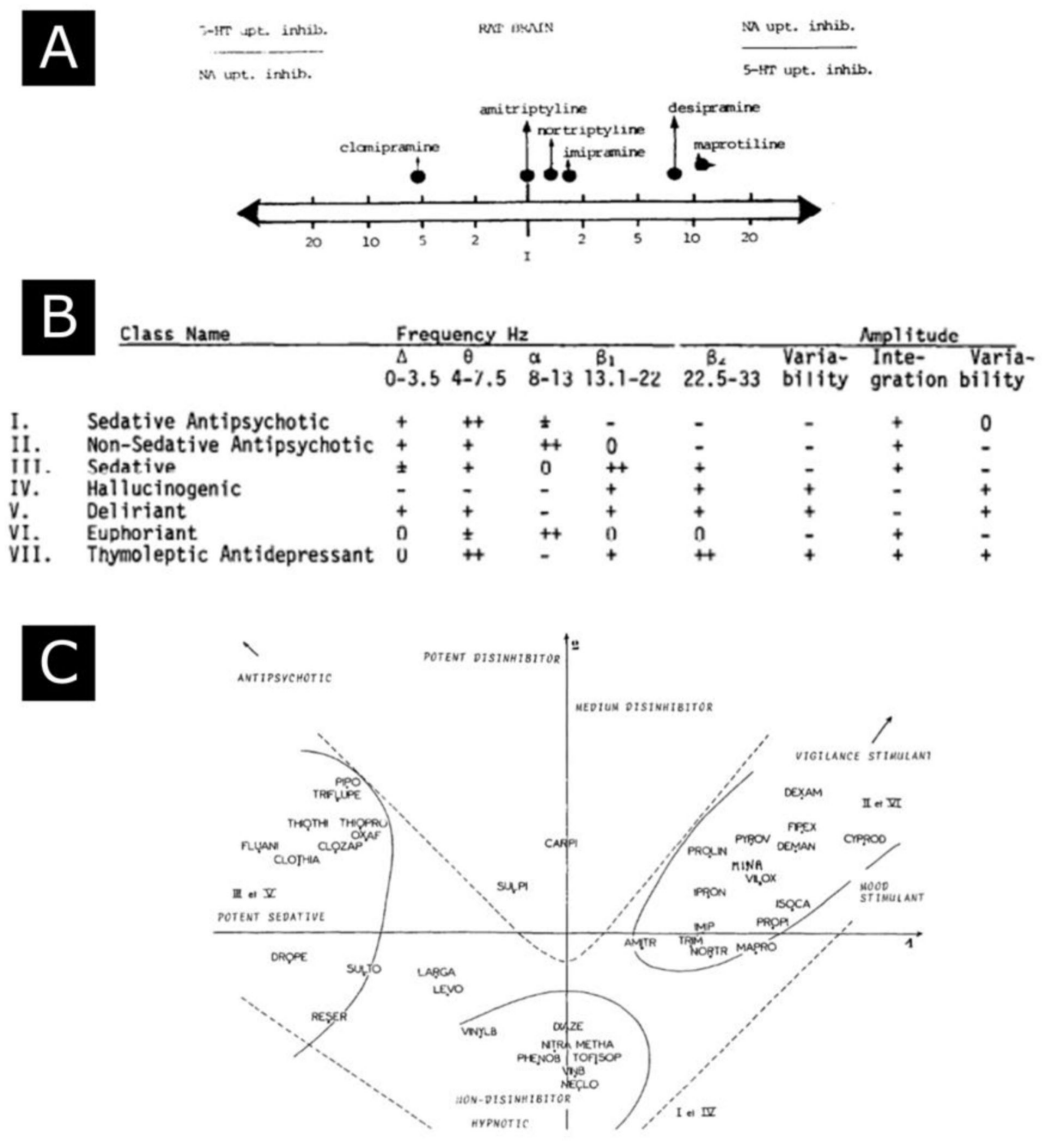
Early attempts at a data driven classification (A) Characterisation of antidepressant in terms or relative potency for inhibiting serotonin and noradrenaline uptake.(16) (B) EEG informed classification.(17) (C) Multifactorial analyses of > 100 compounds on the basis of physiochemical, pharmacological, clinical and toxicological properties identifies 5 axes.(9)

A symptom-based approach is also more compatible with a multi-dimensional view of drug action, where regardless of a focus on pharmacological or clinical effects, psychotropics can be understood as possessing a spectrum of effects. The idea that psychotropics were not magic bullets characterised by their effect on a single target but could be positioned in a multidimensional space, defined either pharmacodynamically or clinically, was attractive to early psychopharmacologists (Figure 1).(9,44,45)

A purely cross-sectional symptom-based approach does, however, have limitations. For example, this would not distinguish between compounds suited to treating low mood in an individual with a history of only low mood, as opposed to an individual with current low mood and a history of manic symptoms. Here, one potential solution might be to incorporate an assessment of the effect upon not only symptoms, but also on symptom dynamics. Another is to recognise the limitations of a purely symptom-based approach, and to consider whether it is instead perhaps possible to identify the latent causal processes that give rise to clinical phenomena, enabling mechanism-guided molecular intervention.(46)

While psychotropic classification has followed psychiatric classification, the relevance of psychopharmacology to diagnostic taxonomies was also highlighted soon after the entry of these compounds to clinical practice.(47) Over 60 years ago the hypothesis that ‘the response affinity of syndromes to specific chemical constituents [could be] the basis for decoding the still mysterious network of pathogenic links and factors’, remains relevant.(48,49) The proposal that response to treatment might be a probe of underlying biology and thereby a means of identifying more biologically homogenous subgroups has, for example, been put forward in response to the findings that in individuals with schizophrenia, raised markers of striatal dopamine function predict clinical response to dopamine antagonists.(50)

### Multi-Level Mechanisms

**Figure 2:**
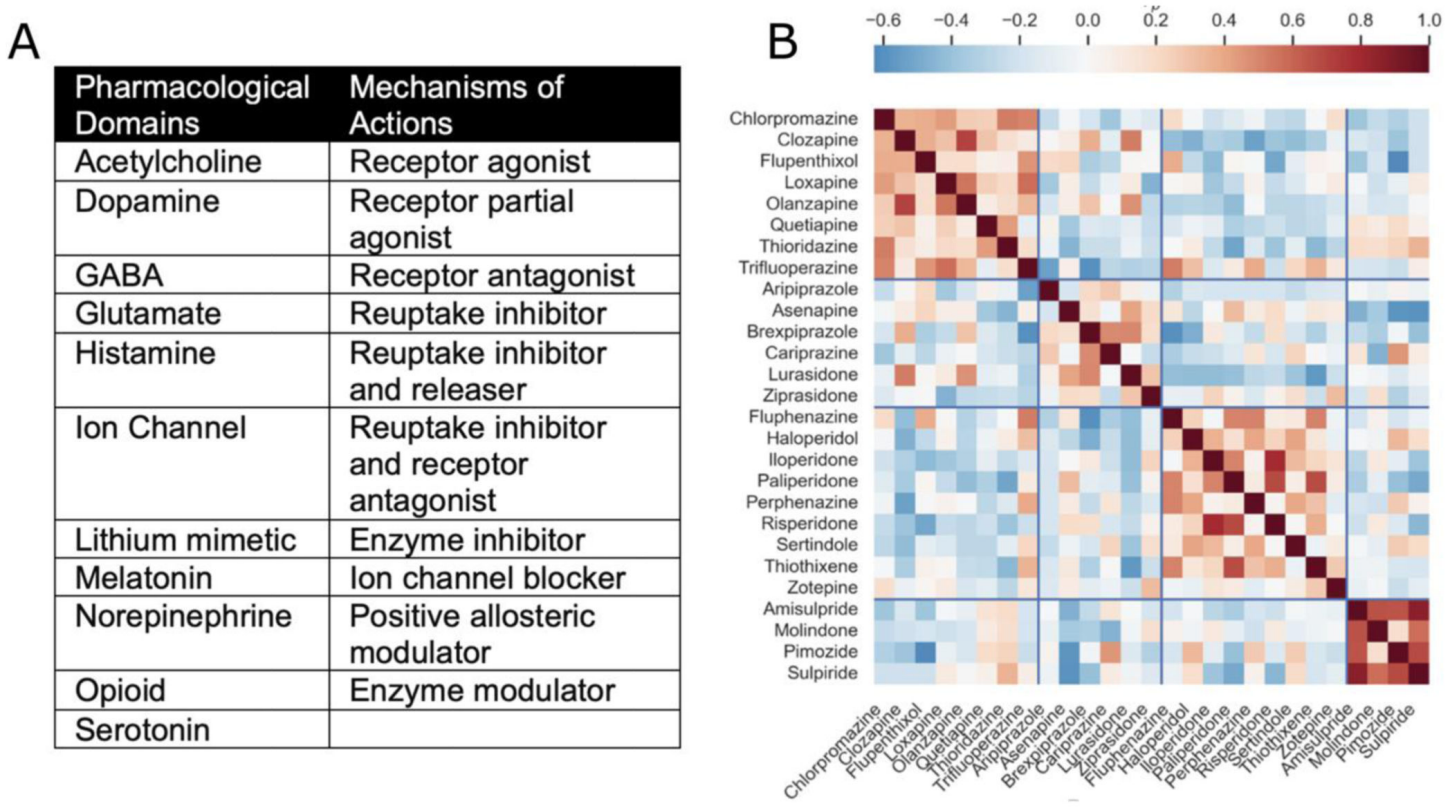
Pharmacodynamically-informed classification schemes (A) The Neuroscience Based Nomenclature classifies compounds according to both neurochemical properties and pharmacodynamic mechanism of action.(23) (B) A data-driven approach to classifying antipsychotic medication, red shading indicates that compounds share a similar receptor binding profile. Compounds with a similar profile are then clustered together.

In addition to understanding psychotropic effects as acting on multiple dimensions, it will also be useful to view effects as existing on multiple levels (Figure 2). The classification schemes discussed above mostly focus on the poles of this multilevel approach, namely molecular level targets at one end and clinical disorders at the other. Even at these extremes there is complexity: for example, different intracellular signalling effects for different 5HT2A receptor agonists at the level of receptor pharmacology, and competing diagnostic classification systems at the clinical level.(51,52) In between these two extremes, however, are mediating levels of neuronal circuits, cognitive/behavioural processes, and symptom level phenomena. Characterising the impact of compounds at these levels has the potential to generate schemes that more meaningfully capture the spectrum of a drug’s effects and inform the situation in which it might be most effective.

This investigation of psychotropics at multiple intervening levels was undertaken soon after compound discovery,(53) with several groups attempting to use electroencephalography and behavioural markers to classify compounds.(17,54,55) It was also apparent that most psychotropic compounds had clinically important effects outside the central nervous system,(56) and indeed considering the impact of treatments on domains such as metabolic health is a crucial part of psychotropic prescribing.(12,57,58)

### Drug Development

Target-based drug development aims to develop compounds acting at proteins or genes known to be causally implicated in pathophysiology or symptom generation. Exemplar cases include the recent successes of gene therapies for monogenetic disorders such as haemophilia A, and targeted immunotherapies for molecularly-characterised cancers.(59,60) Following target identification, *target engagement*, the ascertainment that a compound binds to and affects a target of interest, may increase the likelihood of subsequent success.

Target identification rests on deep understanding of the molecular machinery of disease, often aided by valid preclinical models. This pre-requisite is not yet met for any common psychiatric disorder. In psychiatric contexts, disorders rarely possess a simple one-to-one mapping to molecular substrates, and many existing treatments show a diverse pharmacological binding profile. The complexity of psychiatric pathophysiology makes the identification of single targets challenging. While preclinical models exist, they have generally not proven themselves as valid wellsprings of translatable molecular targets and the mapping to human neurobiology and behaviour may be unclear. As a result, one may demonstrate target engagement (e.g. via either in vivo or ex vivo receptor occupancy studies), but as the links between target and behaviour are typically not definitively established, this may not guarantee clinical efficacy. The development of phosphodiesterase 10A (PDE10A) inhibitors is an example in which target engagement and efficacy in animal models was demonstrated but no efficacy signal was detected in clinical trials.(61)

As a result of these challenges, drug development often involves creating modulators of targets chosen based on the efficacy of existing treatments, which themselves were often discovered through serendipitous clinical observation, rather than mechanism-guided target-based design.

In contrast, phenotypic-based drug development involves observing the downstream effects of compounds (which act at the molecular level) on the functioning of a biological system (often at a more emergent level such as behaviour). In psychiatry, this typically employs quantifying the effects on an animal model of a particular disorder (e.g., modulation of locomotor activity in psychosis). One limitation, however, is that these animal models often become established due to their ability to demonstrate drug effects of existing efficacious treatments, rather than their relevance to human pathophysiology. For example, the ability to abolish amphetamine-induced hyperlocomotion is a common screen for antipsychotic effects. The link between this phenotype and psychosis is, however, weaker than the link between the phenotype and excessive dopaminergic neurotransmission. As a result, this screen may primarily identify modulators of dopamine signalling, rather than drugs with an antipsychotic effect per se.

A innovative approach to phenotypic-based screening was recently employed using automated analysis of video recordings to analyse rodent behaviour in a mechanism-agnostic fashion.(62,63) A broad array of behavioural batteries was used, with a supervised machine learning algorithm used to analyse the behavioural readout, and identify compounds with an antipsychotic like behavioural signature. This identified a TAAR1 agonist as possessing antipsychotic activity, with the compound subsequently entering phase III clinical trials.(64) However, this approach will still possess many of the drawbacks described above, as training data consists of behaviours associated with existing compounds. Consequently, the algorithm is more likely to identify known mechanisms of action rather than novel ones, as the behaviour patterns are linked to already established categories. In the case of TAAR1 agonists, while potentially still a meaningful advance the relevant mechanism of action does still appear to converge upon modulation of striatal dopamine signalling.(65) Partially addressing this concern, data-driven decomposition of complex naturalistic behaviour may have potential to identify clinically-relevant behavioural/cognitive building blocks that can be differentially targeted at the molecular level, thus identifying novel drug targets.(66)

In general, both phenotypic and target-based approaches are biased to refine existing mechanisms of action rather than develop novel compounds or new indications for existing compounds. This relatively narrow approach is also encouraged by regulatory systems where compounds will be evaluated for a disorder specific indication, using focused clinical trial designs with well-established endpoints. As a result of this clinical evaluation, the action of drugs is often defined by what they have been tested for, rather than their broader potential effects.

### A Taxonomy for the 21^st^ Century

**Figure 3:**
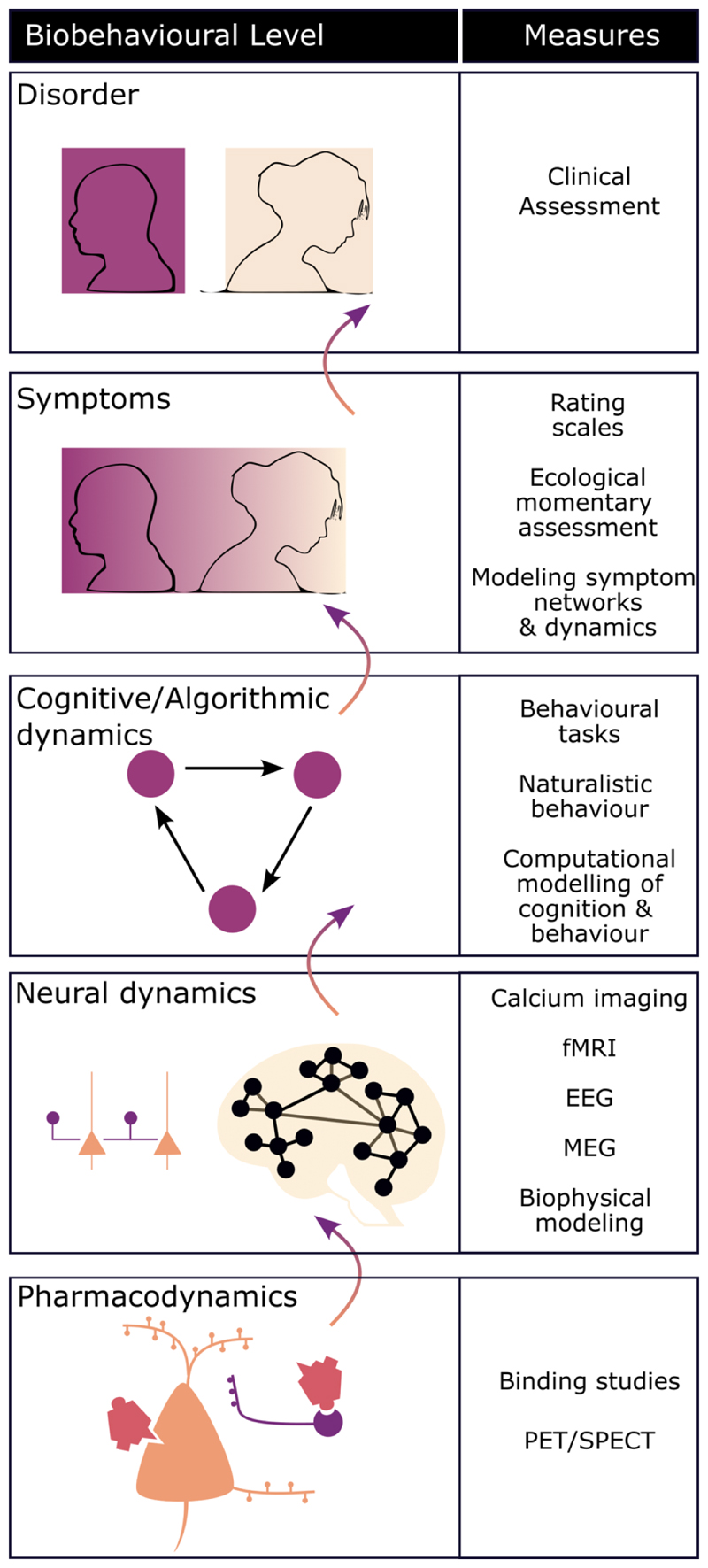
Multiple levels of psychopharmacological action

**Figure 4:**
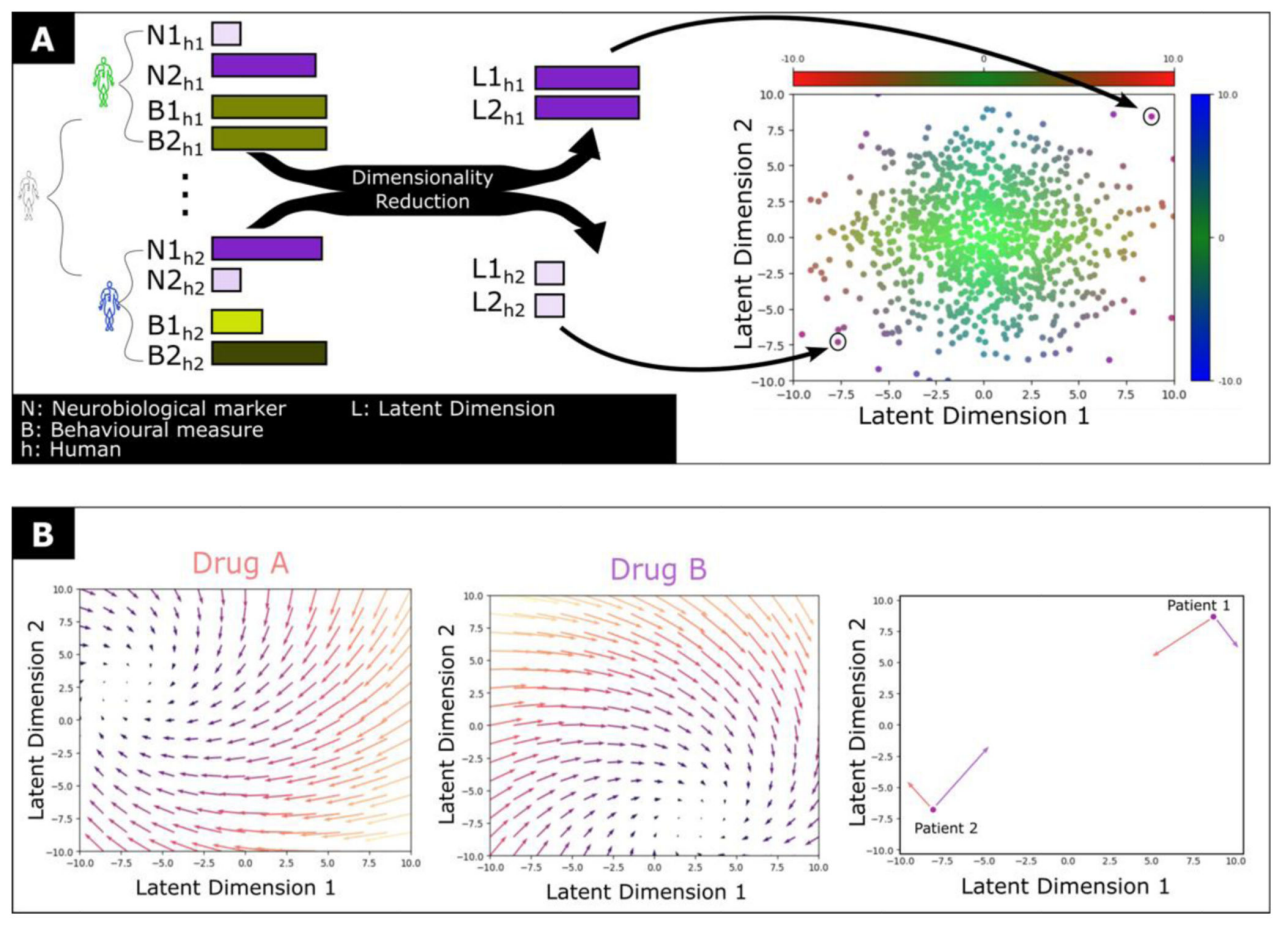
Building a data driven multilevel taxonomy (A) Individuals are characterised across multiple neurobiological and behavioural measures, followed by dimensionality reduction. (B) The effects of psychotropics can be characterised based how they shift individuals across this latent space. In addition to providing a mechanistically meaningful form of classification, this directly supports personalised treatment e.g., Drug A is likely to normalise the neuro/behavioural profile of patient 1, while for patient 2 drug B is more likely to achieve this.

The challenges described above include a diagnostic system in which categories are heterogenous in terms of neurobiology and symptoms. This is compounded by the fact that most interventions possess a non-specific pharmacodynamic profile. We propose that to enable a fundamental advance in psychotropic classification it is necessary to evaluate both molecular effects of psychotropics and clinical effects in patients in the same “representational space”. In practice this means broad and deep phenotyping of clinical cohorts and drug action across multiple levels of description (Figure 3 and Table 1), combined with data-driven dimensionality reduction approaches capable of embedding this high-dimensional information within a shared low-dimensional representational space (Figure 4). Initial work here has remained connected to existing diagnostic categories,(67,68) although there is recognition that a more transdiagnostic approach may have significant benefits.(69,70)

One key question is the extent to which meaningful signal is available that can be extracted in a relatively theory-agnostic (data-driven) fashion. For example, if sufficient clinically relevant variance is inherent within task free functional and structural neuroimaging measures, then this can potentially be extracted via data-driven machine learning approaches given sufficient sample sizes. An alternative is a theory-driven approach that leverages normative computational theories of cognition and behaviour to characterise inter-individual and inter-drug variance in behaviour in terms of latent cognitive variables (i.e., algorithmic model parameters or structure). In theory, this approach may identify behaviourally and clinically meaningful information in a more data-efficient manner than pure data-driven approaches.(71,72) Both approaches, however, face significant challenges in terms of achieving sufficient reliability and accuracy for clinically meaningful use.(73–76)

An additional challenge is that animal models used to develop and characterise psychotropics have an unclear mapping to human biology and symptomatology. As a result, a circular logic develops in which compounds are identified as having potential clinical benefit based on their ability to induce behaviours similar to existing treatments. Building on the approach above, a method to screen compounds centred on their capacity to induce desired shifts in a biobehaviourally meaningful latent space, would be valuable. A necessary step here is the need to develop a cross-species common space for measures of both pathophysiology and psychopharmacology.

**Figure 5:**
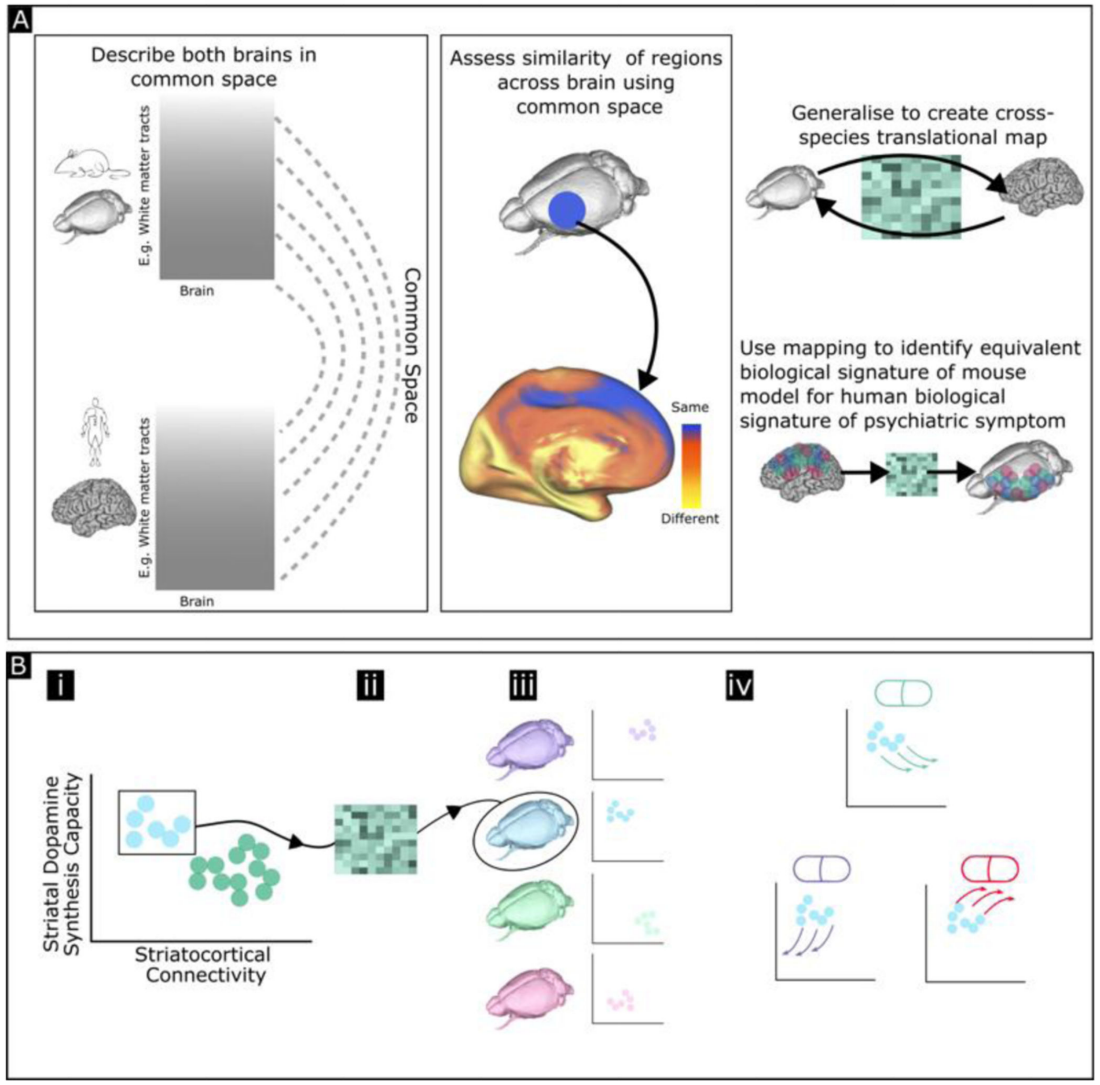
Building a cross-species mapping (A) Developing a mapping to allow translation of brain features between species. (B) Using this mapping in drug development. (i) Profile associated with symptoms or disorder identified in a patient population (blue circles) (ii) Cross species mapping used to generate the analogous profile in e.g., mice (iii) Animal model selected that best recapitulates this profile (iv) This animal model then employed to screen potential compounds and identify that which best moves profile in desired direction (top green compound in this case).

Recent work has focused on developing a mapping that allows a biological space in one species to be translated to that of another.(77,78) In this method brains are described in a common feature space, using features for which cross-species analogues exist. For example, a brain region can be described in terms of its gene expression profile, by restricting to genes with clear cross-species correspondence then brains of different species can be described in a common ‘gene space’; this allows for a mapping from each brain part in one species to that in another (Figure 5). Other common spaces such as structural or functional connectivity are also possible. Other methodological approaches complementary to this aim include more ecologically valid methods of characterising both human and rodent behaviour. The use of actigraphy and natural language in the study of humans (79,80), and automated analyses of naturalistic rodent behaviour, (66) have the potential to enable a pharmacological characterisation that transcends constraints inherent to lower dimensional measures of behaviour.

The development of this common space means both that animal models can be identified that specifically recapitulate biologically meaningful human subtypes, and that it is possible to predict the impact of a compound on a relevant latent space in humans based on its effect on that same space in an animal model.

While the above description has the potential to significantly advance both the identification of novel compounds and repurposing of existing treatments, it is not intended to be presented as a universal solution. For example, clinicians and patients may find benefit in a descriptive overlay in which compounds are described in terms of their symptomatic effects.

## Conclusion

Psychotropic classification has remained relatively unchanged over the past 70 years. To some extent this reflects the clinical utility of existing schemes. It also, however, reflects the lack of progress in developing a pathophysiologically-informed psychiatric diagnostic system, and – relatedly - in developing novel treatments with meaningfully greater efficacy. Psychotropic taxonomies, psychiatric diagnostic systems, and approaches to psychiatric drug development are intertwined, and together resist fundamental change. For meaningful advances a significant re-envisioning of both psychiatric and psychotropic approaches may be required.
